# Differences in sperm protein abundance and carbonylation level in bull ejaculates of low and high quality

**DOI:** 10.1371/journal.pone.0206150

**Published:** 2018-11-14

**Authors:** Agnieszka Mostek, Błażej Westfalewicz, Mariola Słowińska, Mariola Aleksandra Dietrich, Sylwia Judycka, Andrzej Ciereszko

**Affiliations:** Department of Gamete and Embryo Biology, Institute of Animal Reproduction and Food Research, Polish Academy of Sciences in Olsztyn, Tuwima 10, Olsztyn, Poland; Universite Clermont Auvergne, FRANCE

## Abstract

In breeding and insemination centres, significant variation in bull ejaculate quality is often observed between individuals and also within the same individual. Low-quality semen does not qualify for cryopreservation and is rejected, generating economic loss. The mechanisms underlying the formation of low-quality ejaculates are poorly understood; therefore, the aim of the present study was to investigate the proteomic differences and oxidative modifications (measured as changes in protein carbonylation level) of bull ejaculates of low and high quality. Flow cytometry and computer-assisted sperm analysis were used to assess differences in viability, reactive oxygen species (ROS) level, and sperm motility. To analyse changes in protein abundance, two-dimensional difference gel electrophoresis (2D-DIGE) was performed. Western blotting in conjunction with two-dimensional electrophoresis (2D-oxyblot) was used to quantitate carbonylated sperm proteins. Proteins were identified using matrix-assisted laser desorption/ionisation time-of-flight/time-of-flight spectrometry. High quality ejaculates were characterised by higher sperm motility, viability, concentration, and a lower number of ROS-positive cells (ROS^+^). We found significant differences in the protein profile between high- and low-quality ejaculates, and identified 14 protein spots corresponding to 10 proteins with differences in abundance. The identified sperm proteins were mainly associated with energetic metabolism, capacitation, fertilisation, motility, and cellular detoxification. High-quality ejaculates were characterised by a high abundance of extracellular sperm surface proteins, likely due to more efficient secretion from accessory sex glands and/or epididymis, and a low abundance of intracellular proteins. Our results show that sperm proteins in low-quality ejaculates are characterised by a high carbonylation level. Moreover, we identified, for the first time, 14 protein spots corresponding to 12 proteins with differences in carbonylation level between low- and high-quality ejaculates. The carbonylated proteins were localised mainly in mitochondria or their immediate surroundings. Oxidative damage to proteins in low-quality semen may be associated with phosphorylation/dephosphorylation disturbances, mitochondrial dysfunction, and motility apparatus disorders. Our results contribute to research regarding the mechanism by which low- and high-quality ejaculates are formed and to the identification of sperm proteins that are particularly sensitive to oxidative damage.

## Introduction

The success of bovine artificial insemination programs largely depends on the use of high-quality semen that allows the efficient reproductive genetic selection of cattle [1]; however, variability in the quality of bull ejaculates in breeding and insemination centres is often observed [2,3]. The quality of ejaculates from the same bull may vary significantly in terms of sperm concentration, motility, and viability [4], and differences in the motility and content of particular sperm proteins can also be found between sperm populations within the same ejaculate [5]. Low-quality ejaculates that do not fulfill the quality criteria (concentration of at least 1 × 10^9^ sperm/mL and a sperm motility of at least 70%) are disqualified from cryopreservation, which generates economic loss.

Several factors affect ejaculate quality, including breed, age, management factors, body condition, and environmental stresses [4]; however, the mechanisms underlying the formation of low-quality ejaculates are poorly understood. High-throughput techniques such as transcriptomics [6], proteomics [7], and metabolomics [8] provide insight into the molecular mechanisms underlying bull sperm physiology, with reference mainly to differences in bull fertility. Among these molecular levels, proteins appear to be the main effectors of cell functioning [9].

The dynamic development of proteomic techniques has allowed the description of numerous proteins of bull seminal plasma [10,11] and reproductive tract secretions [12,13], in addition to the identification of a number of sperm fertility-related protein markers [14]. Two-dimensional difference gel electrophoresis (2D-DIGE) is a particularly useful technique for use in quantitative approaches, allowing the separation of proteins in different samples on the same gel and eliminating gel-to-gel variability [15]. Recent advances in sperm proteomics, including the use of 2D-DIGE, have enabled the analysis of complex proteomes, which has led to a more comprehensive view of the molecular changes associated with bull sperm maturation [16], cryopreservation [17], and fertility [18]. The use of an advanced proteomic technique such as 2D-DIGE holds promise for the elucidation of the association between the sperm protein profile and the formation of ejaculates of different quality.

OxiProteomics is an innovative proteomics branch specialising in the detection of oxidatively modified proteins. It is well-known that sperm proteins undergo redox modifications that mediate signaling pathways important for sperm physiology. Some oxidative modifications of sperm proteins, including oxidation of thiol groups, are needed to ensure sperm motility and capacitation [19]; however, when reactive oxygen species (ROS) are produced at high concentrations, the established oxidative stress promotes pathological changes to sperm proteins, resulting in decreased semen quality [20]. It has been previously shown that the total protein carbonylation level is elevated in sperm showing decreased quality [21] and fertilizing potential [22]; however, to the best of our knowledge, carbonylated proteins have not yet been identified in relation to fresh bull semen. Therefore, studies investigating the oxidative modifications of bull sperm proteins, including carbonylation, in relation to the quality of ejaculates are required.

Considering the above, the aim of the present study was to characterise the proteomic differences between low- and high-quality bull ejaculates, and to identify sperm proteins that are particularly sensitive to oxidative damage in relation to sperm motility, viability, and the number of ROS-positive cells (ROS^+^). Such information is a prerequisite to improving the understanding of the molecular mechanisms underlying the differences in the quality of bull ejaculates.

## Materials and methods

### Collection of ejaculates and selection criteria for semen quality

**Fig 1:**
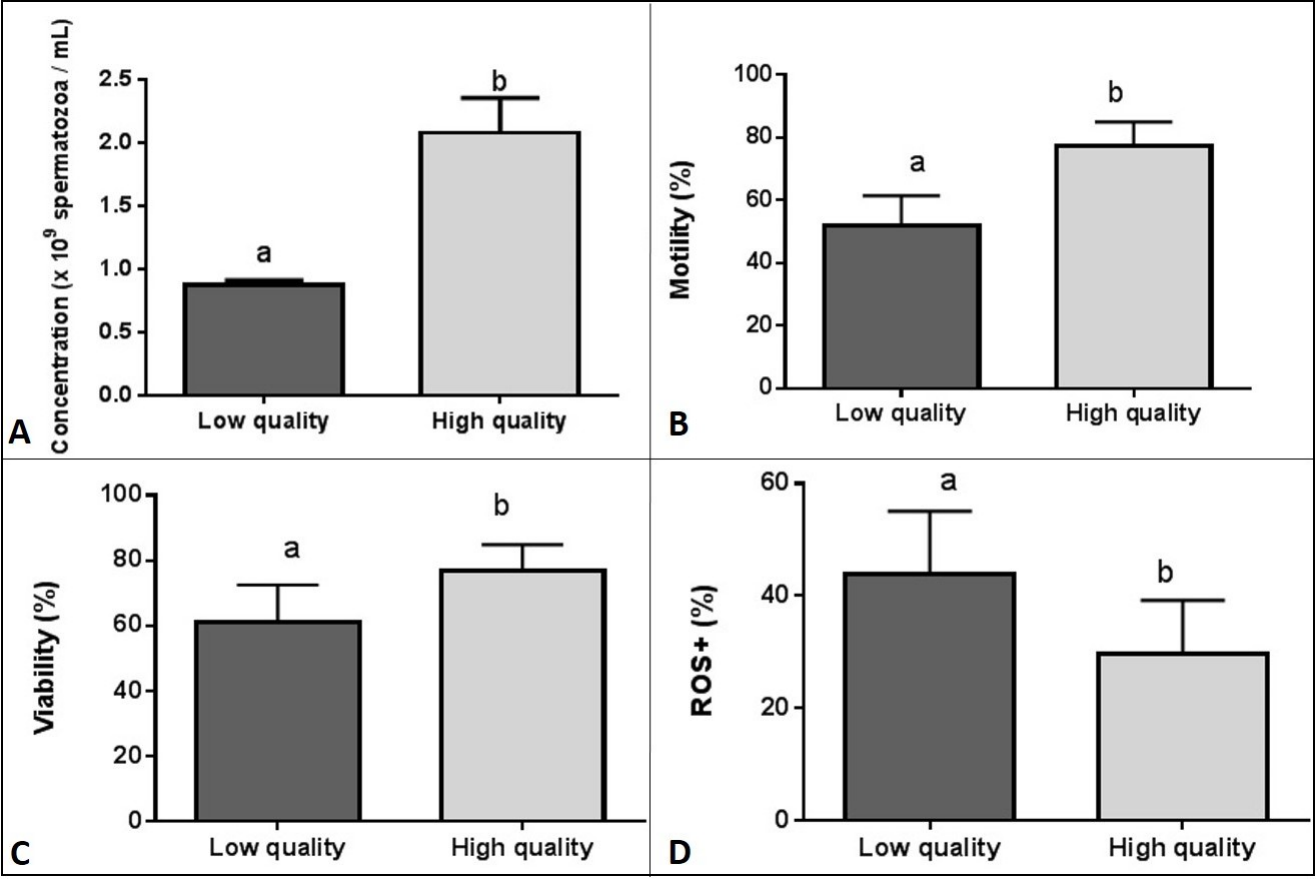
Sperm concentration (A), motility (B), viability (C), and ROS^+^ cells (D) of low- and high-quality bull ejaculates (n = 6 in each group). Results are expressed as the mean ±SD. Different letters indicate significant differences between low- and high-quality ejaculates (*p <* 0.05).

Ejaculates were collected on 13^th^ January 2017 from mature Holstein Friesian bulls maintained at SHiUZ (Animal Breeding and Insemination Centre) in Olecko, Poland (54˚2`N 22˚30`E), using an artificial vagina. The daily feed for each bull consisted of: hay or silage– 8 kg; 18% protein concentrate– 2.5 kg; oats– 1 kg; barley– 0.5 kg; and mineral mix– 100 g. All bulls used in the present study were in good health, as controlled by a veterinary andrologist. Evaluation of bull ejaculate quality was based on sperm motility, concentration, viability, and ROS^+^ content. Among the 19 ejaculates collected by the SHiUZ staff, 12 ejaculates exhibiting opposite quality values were selected; six ejaculates characterised by significantly higher sperm motility (*p* < 0.0001), viability (*p* < 0.05), concentration (*p* < 0.0001), and a lower ROS level (*p* < 0.05) (high-quality ejaculates, HQ) as compared with six ejaculates characterised by lower sperm motility, viability, concentration, and a higher ROS level (low-quality ejaculates, LQ) (Table 1; Fig 1A–1D). No significant differences were found in the movement trajectory between HQ and LQ ejaculates with respect to the following parameters: VCL–curvilinear velocity; VAP–average path velocity; VSL–straight line velocity; BCF–beat cross frequency; or LIN–linearity (S1 Fig).

**Table 1:** Sperm parameters of low- and high-quality ejaculates (n = 6 in each group).

Measured parameter	Low-quality ejaculates (n = 6)	High-quality ejaculates (n = 6)
Concentration [×10^9^ spermatoza/mL]	0.87 (±0.03)***	2.084 (±0.27)
Motility [%]	51.92 (±9.54)***	77.33 (±7.60)
Viability [%]	61.17 (±11.39)*	76.68 (±8.22)
ROS^+^ cells [%]	43.75 (±11.33)*	29.62 (±9.63)

*p < 0.05

***p < 0.00001

### Sperm motility analysis

The sperm motility parameters of the ejaculates were measured using computer-assisted sperm analysis (CASA) as described previously [22]. Video recordings of motile sperm were captured using a microscope with a 4 x negative phase objective and a Sony CCD black-and-white video camera (SPTM108CE). MicroCell 50-μm-deep, two-chamber slides (Conception Technologies, San Diego, USA) were mounted on a heated stage (37°C). Semen samples were diluted in BIOXcell extender (IMV Technologies, L'aigle, France) at a ratio of 1:40 prior to motility measurements. Sperm motility parameters were analysed using a Hobson Sperm Tracker (Hobson Vision Ltd., Baslow, UK) as described previously [23]. Each sample was analysed in duplicate. For further analysis, the percentage of total motile sperm was assessed, in addition to the movement trajectory parameters: VSL, VCL, ALH, VAP, and LIN.

### Flow cytometry analyses of viability and oxidative stress

Flow cytometry analyses of the ejaculates (n = 6 in each group) were performed using the Muse Cell Analyser (EMD Millipore, Billerica, USA) as described previously [23]. Viability and sperm concentration were assessed using Muse Count and Viability Reagent (EMD Millipore). A Muse 7-aminoactinomycin D-based DNA-binding dye that stains cells that have lost membrane integrity, allowing the dye to stain the nucleus of dead and dying cells, was used. This parameter was displayed as viability and was used to discriminate between viable (live cells that do not stain) and non-viable (dead or dying cells that stain). The analyses were performed according to the manufacturer’s protocol with evaluation of 1000 events. Briefly, semen was diluted in phosphate-buffered saline (PBS) to obtain concentrations in the range of 1 × 10^5^ to 1 × 10^7^ sperm/mL. Subsequently, 20 μL each semen suspension was mixed with 380 μL Muse Count and Viability Reagent and incubated for 5 min at room temperature prior to performing Count and Viability Assay.

The ROS levels of HQ and LQ bull ejaculates were measured using a Muse Oxidative Stress Kit (EMD Millipore) based on dihydroethidium, that allows the determination of the count and percentage of cells undergoing oxidative stress according to the intracellular detection of superoxide radicals. The procedure was performed in accordance with the manufacturer’s recommendations with evaluation of 3000 events. Briefly, semen was diluted in assay buffer to obtain concentrations in the range of 1 × 10^6^ to 1 × 10^7^ sperm/mL. Subsequently, 10 μL each semen suspension was mixed with 190 μL Muse Oxidative Stress working solution and incubated for 30 min (37°C). To accurately determine the cut-off point between ROS-positive (ROS^+^) and ROS-negative (ROS^-^) samples, a positive control containing completely oxidised sperm cells was used. The positive control was prepared by incubating the sperm cells in the presence of 2 mM menadione (Sigma-Aldrich, St. Louis, USA), a superoxide anion generator, for 1 h at 37°C.

### 2D-DIGE

To obtain sperm protein extracts, 0.5 mL fresh bull semen samples, representing LQ and HQ ejaculates, were centrifuged at 700 × *g* for 30 min at 4°C to separate sperm cells. The pellets were resuspended in 105 μL lysis buffer containing 7 M urea, 2 M thiourea, 2% 3-[(3-cholamidopropyl)-dimethylammonio]-1-propanesulfonate (CHAPS), 2% [v/v] immobilised pH gradient (IPG) buffer 3–10 NL, 50 mM dithiothreitol (DTT), and 0.5% (v/v) protease inhibitor cocktail (Sigma-Aldrich). The samples were sonicated on ice six times for 5 s at 30% amplitude, and subsequently centrifuged at 10000 × *g* for 60 min at 4°C. Protein concentration was measured using the Bradford assay (Coomassie Plus Assay Reagent, Thermo Fisher Scientific). Protein extracts were used to perform proteomic analyses.

Fluorescence labelling of samples with CyDyes and the 2D-DIGE procedure were carried out according to previously described methodology [16]. Briefly, an internal standard was created by mixing equal amounts of proteins from all samples. Sperm protein samples, along with the internal standard, were labelled with Cy2, Cy3, and Cy5 DIGE Fluor Minimal Dyes (GE Healthcare, Uppsala, Sweden). Samples were loaded onto 24-cm Immobiline DryStrips, with a 3−10 nonlinear gradient pH range (GE Healthcare), and rehydrated for 12 h. Afterwards, the proteins were separated by isoelectric focusing using an Ettan IPGphor apparatus (GE Healthcare). Subsequently, isoelectric focusing strips were equilibrated and transferred to precast 2D-DIGE Ettan DALT Gels (GE Healthcare) as described previously [17]. The obtained gels were scanned using a Typhoon 9500 FLA scanner (GE Healthcare) and analysed using the DeCyder Differential In Gel Analysis software version 5.02 (GE Healthcare) to identify differences in the fluorescence intensities of the spots from LQ and HQ ejaculates. The gels were then stained with colloidal Coomassie Brilliant Blue (CBB) G-250, and spots were manually cut out from the gels for mass spectrometry identification.

### Detection of oxidised proteins

Sperm protein extracts, containing 150 μg protein, were obtained from LQ and HQ ejaculates (as described in the previous paragraph) and derivatised with a four-fold volume of 2,4-dinitrophenylhydrazine (DNPH) solution or derivatisation-control solution (Oxyblot Protein Oxidation Detection Kit, EMD Millipore). The protein samples were purified using a Clean-Up Kit (GE Healthcare) according to the manufacturer’s protocol. The final pellet was dissolved in rehydration buffer (7 M urea, 2 M thiourea, 2% CHAPS, 2% [v/v] IPG buffer 3–10 NL, 50 mM DTT, and bromophenol blue). Insoluble components were removed by centrifugation at 10000 × *g* for 5 min. Protein concentration was measured using the Bradford assay (Coomassie Plus Assay Reagent, Thermo Fisher Scientific).

Samples containing 100 μg protein were loaded onto 7-cm Immobiline DryStrips, with a 3–10 nonlinear pH range (GE Healthcare), and rehydrated for 12 h. Proteins were separated by isoelectric focusing using an Ettan IPGphor apparatus (GE Healthcare) at 20°C, with the current limited to 50 μA per strip and the voltage program as described previously [23]. The IPG strips underwent equilibration and electrophoretic separation performed on 12.5% sodium dodecyl sulfate polyacrylamide gels as described previously [23]. The control gels treated earlier with derivatisation-control solution were stained with CBB and scanned at 300 dpi using an Image-Scanner III (GE Healthcare).

The gels containing DNPH-derivatised proteins were transferred to polyvinylidene fluoride membrane (15 V/gel for 2 h) and subsequently blocked for 1 h with 1% bovine serum albumin in PBS-T buffer (PBS with 0.5% [v/v] Tween-20). Oxyblots were incubated with a rabbit anti-dinitrophenyl (DNP) primary antibody (1:150) overnight at 4°C (Oxyblot™ Protein Oxidation Detection Kit, EMD Millipore). Membranes were washed three times with PBS-T buffer and incubated for 1 h with an anti-rabbit IgG horseradish peroxidase (HRP)-conjugated secondary antibody diluted 1:300 (Oxyblot™ Protein Oxidation Detection Kit, EMD Millipore), and developed as described previously [23].

### Analysis of two-dimensional gel images and oxyblots

Comparative analysis of gels and blots of LQ and HQ ejaculates was performed using the SameSpots software (TotalLab, Newcastle, UK) as described previously [24]. Spot intensities were automatically normalised to the total density of spots on the corresponding gel or blot to correct for slight differences in staining and/or loading. Normalised protein spots that showed changes (*p* < 0.05) in carbonylation level on oxyblots were subsequently matched with the corresponding proteins on gels to verify whether there were quantitative changes at the protein level. Only the spots that showed changes on oxyblots and did not show intensity changes on gel replicates were considered as changed carbonylation levels. The spots showing differences in protein carbonylation between LQ and HQ ejaculates were manually cut from the gel and subsequently subjected to digestion and protein identification using mass spectrometry.

### Protein digestion and MALDI-TOF/TOF protein identification

Gel plugs were washed, destained, and digested as described previously [24]. Briefly, the protein spots were digested overnight at 37°C using a trypsin solution (Promega, Madison, USA). The peptide mixtures were desalted using ZipTip C-18 RP tips (Millipore, Billerica, MA, USA). Peptide samples were analysed using matrix-assisted laser desorption/ionisation time-of-flight/time-of-flight spectrometry (MALDI-TOF/TOF) on an Autoflex mass spectrometer (Bruker Daltonics, Bremen, Germany). The MS spectra, together with the MS/MS spectra, were searched using the Mascot Server (Matrix Science, London, UK) of the National Centre for Biotechnology Information *Bos Taurus* database, with search parameters as described previously [24]. Matches considered statistically significant (*p <* 0.05) by Mascot, with at least two correctly identified parent ions, were regarded as correct hits.

### Gene ontology analysis

Gene ontology (GO) annotation was used to determine the function and cellular localisation of the proteins in our dataset. The GI numbers of the identified proteins that showed differences in protein abundance and carbonylation were matched to the UniProtKB database to obtain GO annotation using the categories “molecular function” and “cellular component”. With respect to proteins for which the functions and locations were specific for sperm cells, literature references are given.

### Statistical analysis

The statistical analyses of sperm motility, viability, concentration, and ROS^+^ cells in LQ and HQ ejaculates were performed using a Student's *t*-test at a significance level of 0.05 in the GraphPad Prism software (GraphPad Software Inc., San Diego, USA), as described previously [24]. Statistical analysis of the changes in protein abundance was performed using the Biological Variance Module of the DeCyder Differential In Gel Analysis software version 5.02 (GE Healthcare) in six biological replicates (individual ejaculates). The data are expressed as log standardised abundance to ensure a normal distribution. Experimental groups were compared using a Student's *t*-test. Changes in protein spot abundance were considered statistically significant at *p* < 0.05, with a fold change of ±1.2. Normalised spot intensities on oxyblots were compared between treatments using a Student's *t*-test at a significance level of 0.05, using the SameSpots software as described previously [24].

## Results

### Identification of proteins showing changes in abundance

**Fig 2:**
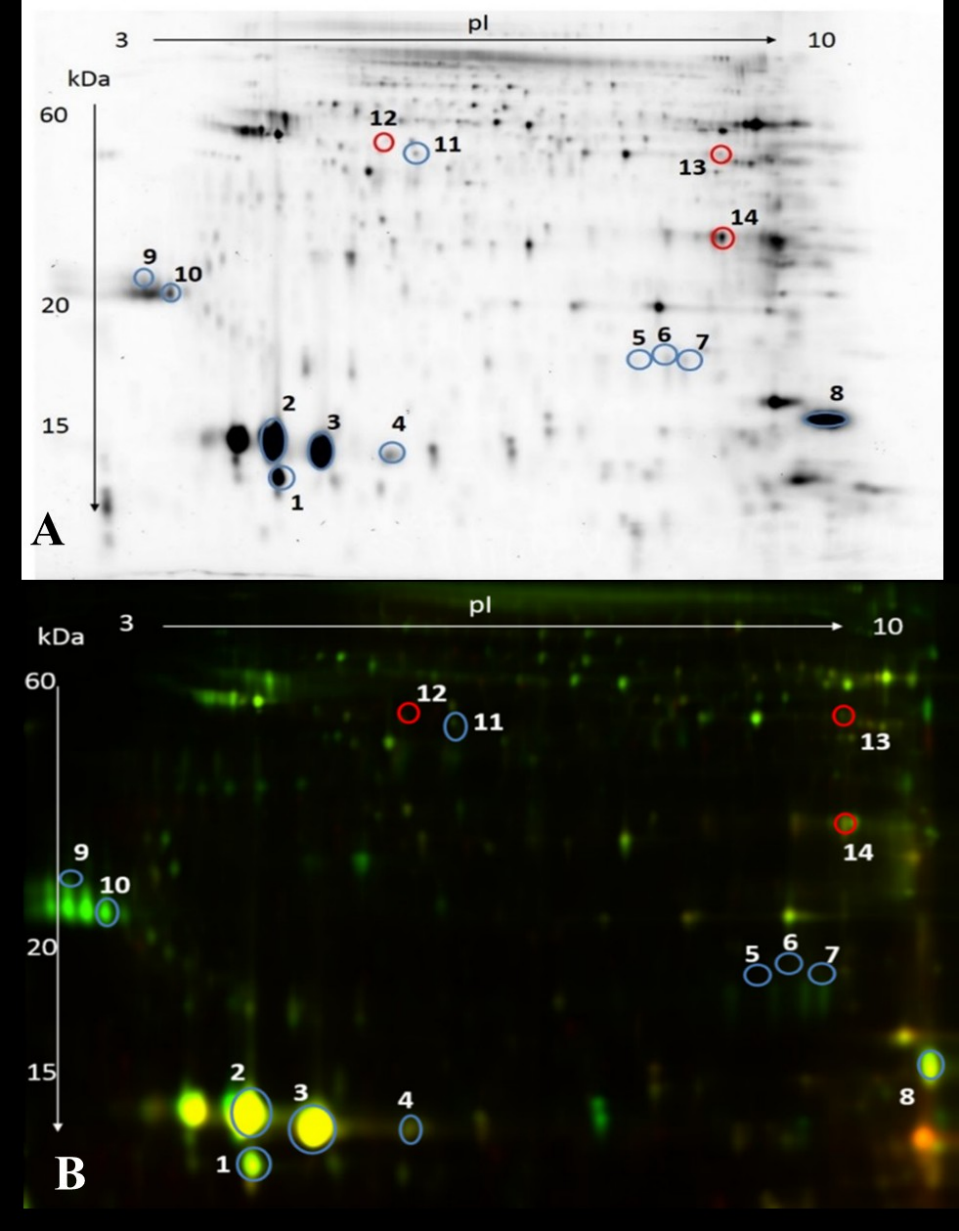
Two-dimensional differential electrophoresis gels showing the proteomic profiles of sperm from HQ and LQ bull ejaculates. Blue protein spots show a significant (*p <* 0.05) increase in abundance in HQ ejaculates and red protein spots show an increase in abundance in LQ ejaculates. The marked protein spots are numbered and listed in Table 2. Single channel representative image (A); and fluorescent overlay view (B).

We isolated and identified 14 protein spots corresponding to 10 proteins in sperm extracts that showed a significant difference in abundance between LQ and HQ ejaculates (*p <* 0.05) (Fig 2; Table 2). Among the 14 identified protein spots, 11 showed an increased abundance in HQ ejaculates, including two proteoforms of binder of sperm protein 1 (BSP1), binder of sperm protein 5 (BSP5), glutathione S-transferase mu 1 (GST), and a single proteoform of spermadhesin 1 (SPADH 1), spermadhesin Z13 (SPADH 13), superoxide dismutase (SOD), adenylate kinase isoenzyme 1 (AK 1), and mitochondrial succinyl-CoA ligase subunit beta (SUCB). Conversely, three protein spots showed an increased abundance in LQ ejaculates, including two proteoforms of mitochondrial cytochrome b-c1 complex (UCRI) and a single proteoform of L-lactate dehydrogenase C isoform (LDHC).

### GO analysis of differentially abundant proteins in LQ and HQ ejaculates

The sperm proteins that showed differences in abundance between LQ and HQ ejaculates are involved in energetic metabolism (five protein spots), capacitation (four protein spots), cellular detoxification (three protein spots), and fertilisation (two protein spots) (Table 2). The identified proteins were observed as extracellular sperm surface proteins, plasma membrane proteins, extracellular fluid proteins (eight protein spots), and intracellular proteins in the mitochondria, cytoskeleton, and cytoplasm (six protein spots) (Table 2).

**Table 2:** Proteins exhibiting significant differences in abundance in LQ ejaculates as compared with HQ ejaculates (n = 6 in each group) (*p <* 0.05). Up-arrows indicate protein spots with increased abundance in HQ ejaculates, while down-arrows indicate protein spots with increased abundance in LQ ejaculates.

Spot no.	Protein name	GI number	Sequence coverage(%)	Protein score	Ionscore > 30	Function in sperm cells	Cellularlocalisation	Ratio
1↑	Spermadhesin 1	157833803	81	123	2	Fertilisation	Cell surface(seminal plasma protein)	-4.2
4↑	Spermadhesin Z13	126158907	53	389	3	Fertilisation	Cell surface(seminal plasma protein)	-2.8
2↑	Binder of sperm protein 1	20663780	52	349	2	Capacitation	Cell surface(seminal plasma protein)	-2.1
3↑	Binder of sperm protein 1	20663780	21	148	1	Capacitation	Cell surface(seminal plasma protein)	-2.0
9↑	Binder of sperm protein 5	28849953	19	229	3	Capacitation	Cell surface(seminal plasma protein)	-4.0
10↑	Binder of sperm protein 5	28849953	24	148	2	Capacitation	Cell surface (seminal plasma protein)	-3.4
12↓	Cytochrome b-c1 complex, mitochondrial	3891848	54	281	2	Energetic metabolism	Mitochondria	1.2
13↓	Cytochrome b-c1 complex, mitochondrial	3891848	43	324	4	Energetic metabolism	Mitochondria	1.2
8↑	Adenylate kinase isoenzyme 1	61888850	68	207	2	Energetic metabolism	Cytoskeleton	-4.6
11↑	Succinyl-CoA ligase subunit beta, mitochondrial	77736229	36	276	2	Energetic metabolism	Mitochondria	-1.2
14↓	L-lactate dehydrogenase C isoform 1	296471858	61	278	2	Energetic metabolism	Mitochondria	1.3
						Fertilisation	Plasma membrane	
							Cytoplasm	
5↑	Superoxide dismutase	88853816	39	240	2	Cellular detoxification	Mitochondria	-6.3
							Cytoplasm	
							Extracellular fluid	
6↑	Glutathione S-transferase mu 1	114053087	80	723	7	Cellular detoxification	Plasma membrane	-5.5
						Fertilisation		
7↑	Glutathione S-transferase mu 1	114053087	40	343	3	Cellular detoxification	Plasma membrane	-6.1
						Fertilisation		

### Identification of carbonylated proteins

**Fig 3:**
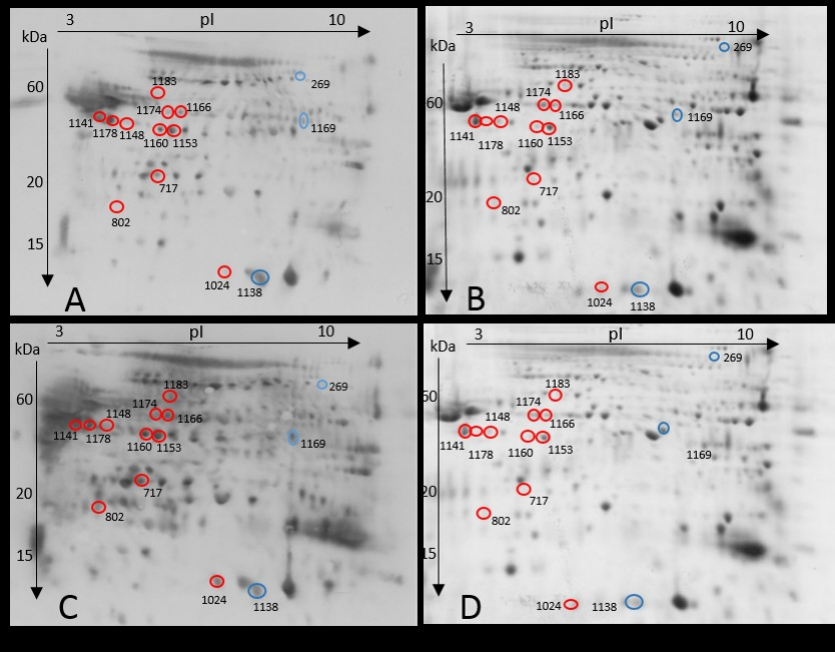
Representative two-dimensional (2D) gels and oxyblots of LQ and HQ ejaculates. 2D oxyblot showing carbonylated proteins in HQ (A) and LQ ejaculates (C). 2D gel stained with Coomassie Brilliant Blue showing the total proteins in HQ (B) and LQ ejaculates (D). Differentially carbonylated protein spots are numbered and listed in Table 3. The protein spots marked in red indicate higher carbonylation in LQ ejaculates while protein spots marked in blue indicate higher carbonylation in HQ ejaculates.

We identified 14 protein spots corresponding to 12 proteins that showed a significant difference in carbonylation level between LQ and HQ ejaculates (*p* < 0.05; Fig 3; Table 3). Of these 14 identified proteins, 11 showed increased carbonylation in LQ ejaculates, including two proteoforms of mitochondrial ATP synthase H^+^-transporting F1 complex beta subunit (ATP 5B) and UCRI, and single proteoforms of serine/threonine-protein phosphatase PP1 gamma catalytic subunit (PP-1γ), outer dense fibre protein 2 (ODF2), platelet-activating factor acetylhydrolase precursor (PAFAH), GST, cAMP-dependent protein kinase (PKA), glycerol kinase 2 (GK2), and clusterin preproprotein (CLU). Conversely, three protein spots showed increased carbonylation in HQ ejaculates; 5'-nucleotidase (NT5), spermadhesin 1 (SPZ1), and metalloproteinase inhibitor 2 (TIMP 2).

### GO analysis of carbonylated spots

Functional analysis showed that spermatozoa proteins exhibiting differences in carbonylation level between LQ and HQ ejaculates are involved in sperm motility (five protein spots), energetic metabolism (four protein spots), fertilisation (two protein spots), lipid metabolism (one protein spot), metal ion binding (one protein spot), and chaperoning (one protein spot) (Table 3). The identified proteins were observed to be plasma membrane and cell surface proteins (seven protein spots) and intracellular proteins in the mitochondria and cytoskeleton (seven protein spots) (Table 3).

**Table 3:** Proteins exhibiting significant differences in protein carbonylation in LQ ejaculates as compared with LQ ejaculates (n = 6 in each group) (*p <* 0.05). Up-arrows indicate protein spots with increased carbonylation in HQ ejaculates, while down-arrows indicate protein spots with increased carbonylation in LQ ejaculates.

Spot no.	Protein name	GI number	Sequence co coverage(%)	Protein score	Ionscore > 30	Function in sperm cells	Cellularlocalisation	Ratio
717 ↓	Serine/threonine-protein phosphatase PP1-gamma catalytic subunit, partial	946618993	31	331	3	Motility	Mitochondria	1.8
							Nucleus	
1166 ↓	Outer dense fibre protein 2	296482070	33	346	4	Motility	Cytoskeleton	1.3
							Cillium	
1183 ↓	Platelet-activating factor acetylhydrolase precursor	99028959	41	431	4	Motility	Cell surface (seminal plasma protein)	1.2
1148 ↓	cAMP-dependent protein kinase	115496662	36	318	2	Motility	Mitochondria	2.2
						Capacitation	Cytoskeleton	
							Cell surface (seminal plasma protein)	
1169 ↑	5'-nucleotidase	296482292	42	242	2	Motility	Cell surface(seminal plasma protein)	-1.7
						Capacitation	Plasma membrane	
1024 ↓	Glutathione S-transferase mu 3	114053087	74	313	2	Fertilisation	Plasma membrane	1.9
						Cellular detoxification		
1141 ↓	Mitochondrial ATP synthase, H^+^-transporting F1 complex beta subunit, partial	927129815	55	495	5	Energetic metabolism	Mitochondria	1.3
1178 ↓	Mitochondrial ATP synthase, H^+^-transporting F1 complex beta subunit, partial	89574051	57	417	5	Energetic metabolism	Mitochondria	1.2
1153 ↓	Mitochondrial cytochrome Bc1	37926997	46	377	3	Energetic metabolism	Mitochondria	1.5
1160 ↓	Mitochondrial cytochrome Bc1	37926997	45	293	3	Energetic metabolism	Mitochondria	1.5
1174 ↓	Glycerol kinase 2	375065854	20	168	2	Lipid metabolism	Mitochondria	1.4
269 ↑	Spermadhesin 1	296488313	28	434	6	Fertilisation	Cell surface(seminal plasma protein)	-2.1
1138 ↑	Metalloproteinase inhibitor 2	676282127	32	289	3	Metal ion binding	Cell surface(seminal plasma protein)	-3
802 ↓	Clusterin preproprotein	27806907	27	225	2	Chaperone	Cell surface(seminal plasma protein)	2.1

## Discussion

HQ bull ejaculates were characterised by a higher sperm concentration, motility, and viability, and a lower number of ROS^+^ cells. Sperm obtained from HQ ejaculates displayed a different proteomic pattern to those obtained from LQ ejaculates. Nine proteins showed a higher abundance in HQ ejaculates, and four proteins showed a higher abundance in LQ ejaculates. LQ ejaculates exhibited a higher carbonylation level of nine proteins, and three proteins showed an increased carbonylation level in HQ ejaculates.

Our results indicate that HQ ejaculates are characterised by a higher abundance of extracellular sperm surface proteins (BSP1, BSP5, SPADH1, and SPADH Z13), that bind to sperm following ejaculation [11,25]. There may be two possible reasons for an increased abundance of extracellular proteins: more efficient secretion of these proteins from accessory sex glands and/or epididymis or a greater binding capacity of the proteins to sperm phospholipids. Considering that HQ ejaculates showed a significantly higher concentration of seminal plasma proteins (S2 Fig), more efficient secretion of extracellular proteins from accessory sex glands and/or epididymis appears to be the main cause of the increased extracellular protein abundance, which of course needs to be confirmed in future studies. The extracellular proteins identified in our study (BSP and SPADH) have been previously reported as indicators of bull fertility [7,26]. These proteins are adsorbed on the sperm surface and play an important role in capacitation, formation of the oviductal sperm reservoir, and interaction with the *zona pellucida* [27,28]. These results strongly suggest that the production of HQ ejaculates is related to intensive secretion of proteins from accessory sex glands and/or epididymis and their efficient binding to the sperm surface, which enables capacitation, formation of the sperm reservoir, and proper gamete recognition.

Our results show that LQ ejaculates are characterised by a higher abundance of intracellular proteins such as UCRI and LDHC, which are localised mainly in the mitochondria and fibrous sheath. Since sperm are transcriptionally inactive, a higher abundance of intracellular proteins is likely a result of the formation of new proteoforms due to post-translational modifications (PTMs) [29]. Alteration in the pattern of PTMs depends mainly on the nature of the modifying substances, such as metabolites and free radicals. The increased ROS content recorded in LQ ejaculates can lead to oxidative protein modifications, such as sulfenylation, sulfinylation, sulfonylation, S-nitrosylation, and carbonylation, and accumulation of oxidatively damaged proteoforms [30]. Our results show that the intracellular protein, UCRI, accumulated in LQ ejaculates and was also carbonylated (Table 3). Since protein carbonylation is a hallmark of oxidative stress, it is possible that another oxidative modifications of proteins may accompany the production of LQ ejaculates. In our opinion, the increased abundance of intracellular protein spots in LQ ejaculates may be associated with the accumulation of proteoforms that have lost activity [31], potentially leading to infertility and several sperm abnormalities [32,33].

Phosphorylation/dephosphorylation processes are likely the most important post-translational features of mature mammalian sperm, crucial for motility and the capacitation process [34]. In our opinion, an increased carbonylation of the kinases and phosphatases, PKA, PP-1γ, and GK2, observed in LQ ejaculates, may lead to abnormal phosphorylation/dephosphorylation of sperm proteins. GK2 and PP-1γ are key proteins involved in energetic metabolism and the regulation of sperm motility [35,36]. However, among the identified proteins, PKA is the major player responsible for the activation of sperm tyrosine kinases (with serine and threonine residues) to trigger a cascade of protein phosphorylation involved in sperm motility and capacitation. PKA induces phosphorylation of axonemal dynein, leading to the consumption of ATP, and thus, increases in pH. During capacitation, the PKA regulatory subunit binds to the AKAP proteins, promoting an increase in tyrosine phosphorylation of sperm proteins by the indirect activation of tyrosine kinases [37]. Sperm with a deficiency in tyrosine phosphorylation do not capacitate properly, leading to male infertility. In view of this knowledge, our results suggest that disorders of sperm protein phosphorylation caused by oxidative damage may be an important cause of LQ ejaculates. Therefore, future research should address the detection of disturbances in protein phosphorylation in LQ ejaculates combined with oxidative damage detection of sperm kinases and phosphatases.

Our results show that LQ semen was characterised by an elevated ROS level accompanied by a higher content of carbonylated proteins. In mammalian sperm, ROS are normally generated at low levels by the mitochondria and plasma membrane to perform physiological processes such as capacitation and acrosome reaction [38]; however, an excessive amount of ROS can cause oxidative damage of macromolecules, including proteins. The reasons for the excessive amount of ROS in sperm cells may be both an intensive ROS production and a decrease in the activity of mitochondrial-linked mechanisms involving the elimination and destruction of ROS [39]. The latter suggestion is supported by our results indicating carbonylation of GST in LQ ejaculates, which may lead to disturbances in ROS elimination mechanisms.

Our results show that almost all proteins exhibiting higher carbonylation levels in LQ semen were localised intracellularly, mainly in the inner (UCRI, ATP 5B) and outer (GK2, PP-1γ, PKA) mitochondrial membrane or in the immediate surroundings of mitochondria (ODF2). UCRI and ATP 5B are involved in electron transfer and proton gradient formation, respectively. In turn, PKA, GK2, and PP-1γ are testis-specific isoforms of enzymes involved in sperm motility and flagella development [35,36,40]. Moreover, ODF 2 is a component of the outer dense fibres of the main cytoskeletal structure of the sperm tail, and carbonylation of this protein was also observed in our previous study in cryopreserved bull semen [23]. Oxidative damage of the above proteins can lead to inefficient ATP production and impaired mitochondrial function and flagella morphology [35,41]. In conclusion, our results suggest that the production of LQ ejaculates is associated with elevated ROS levels, which cause the increased carbonylation of mitochondrial and mitochondria-adjacent cytoskeletal proteins, and eventually may affect mitochondrial and flagella function, which may explain the poor motility of sperm in LQ ejaculates. In addition to intracellular proteins, changes in protein carbonylation of surface proteins were also observed in both LQ and HQ ejaculates. This suggests that the above proteins or their proteoforms are particularly susceptible to oxidation. Carbonylation of GST and CLU, which play a protective role in sperm cells, was increased in LQ ejaculates. CLUs are extracellular chaperones that prevent sperm cells from aggregating and precipitating under stress conditions. Moreover, they play an important role in the uptake of hydrophobic peptides during spermatogenesis. As reviewed by Dun et al., (2012) [42], sperm chaperones play key roles in controlling both the morphological transformation of germ cells during spermatogenesis and the post-testicular maturation of these cells as they transit the male and female reproductive tracts. Membrane-bound GST detoxifies sperm cells from electrophilic compounds and is responsible for gamete recognition [43]. The carbonylation of these proteins can result in reduced protective abilities, which can be related to poor plasma membrane integrity, as indicated by a lower viability than HQ ejaculates. It is known that in addition to mitochondria, ROS production also occurs via the sperm plasma membrane NADPH oxidase system [41]. It is possible that the plasma membrane ROS production contributes to carbonylation of proteins bound to the plasma membrane, such as CLU, GST, NT5, and SPZ1, that were identified in the present study. Further studies are necessary to identify the source of ROS involved in the carbonylation of surface proteins and to identify the mechanism responsible for the increased sensitivity of these proteins to oxidation.

In summary, our results suggest that the production of HQ ejaculates is related to efficient secretion of proteins from accessory sex glands and/or epididymis, including sperm surface-binding proteins. LQ ejaculates appear to accumulate specific (probably defective) proteoforms of intracellular proteins. Oxidation of sperm proteins appears to be related to their close localisation to ROS production sites (the mitochondria and plasma membrane). Oxidative damage to proteins in LQ semen may be associated with phosphorylation/dephosphorylation disturbances, mitochondrial dysfunction, and motility apparatus disorders. The proteins identified in the present study can be potential markers of semen quality and ejaculate suitability for cryopreservation. Further studies should be designed to identify protein differences within good quality samples and poor fertility results.

## Supporting information

S1 Fig Motility parameters of LQ and HQ semen (n = 6 in each group). Results are expressed as the mean +SD. No significant differences in the movement trajectory were found between LQ and HQ semen. VCL − curvilinear velocity; VAP − average path velocity; VSL − straight line velocity; BCF − beat cross frequency; and LIN − linearity.
(TIF)

S2 Fig Seminal plasma protein concentrations in LQ and HQ ejaculates. Results are expressed as the mean +SD. Different letters indicate significant differences (*p <* 0.05).
(TIF)

## References

[1] Oliveira LZ, de Arruda RP, de Andrade AFC, Celeghini ECC, dos Santos RM, Beletti ME, et al Assessment of field fertility and several in vitro sperm characteristics following the use of different Angus sires in a timed-AI program with suckled Nelore cows. Livest Sci 2012;146:38–46. 10.1016/j.livsci.2012.02.018

[2] Sudano MJ, Crespilho AM, Fernandes CB, Junior AM, Papa FO, Rodrigues J, et al Use of bayesian inference to correlate in vitro embryo production and in vivo fertility in zebu bulls. Vet Med Int 2011;2011:436381 10.4061/2011/436381 21547211 10.4061/2011/436381 PMC3087428

[3] Correa JR, Pace MM, Zavos PM. Relationships among frozen-thawed sperm characteristics assessed via the routine semen analysis, sperm functional tests and fertility of bulls in an artificial insemination program. Theriogenology 1997;48:721–31. 10.1016/S0093-691X(97)00296-3 16728166 10.1016/s0093-691x(97)00296-3

[4] Barth AD, Waldner CL. Factors affecting breeding soundness classification of beef bulls examined at the Western College of Veterinary Medicine. Can Vet J 2002;43:274–84. 11963661 PMC339235

[5] Netherton J, Hetherington L, Ogle RA, Velkov T, Baker M. Proteomics analysis of good and poor quality human sperm demonstrates several proteins are routinely aberrantly regulated. Biol Reprod 2018;99:395–408. 10.1093/biolre/iox166 29228106 10.1093/biolre/iox166

[6] Feugang JM, Rodriguez-Osorio N, Kaya A, Wang H, Page G, Ostermeier GC, et al Transcriptome analysis of bull spermatozoa: Implications for male fertility. Reprod Biomed Online 2010;21:312–24. 10.1016/j.rbmo.2010.06.022 20638337 10.1016/j.rbmo.2010.06.022

[7] Kumar P, Kumar D, Singh I, Yadav PS. Seminal Plasma Proteome: Promising Biomarkers for Bull Fertility. Agric Res 2012;1:78–86. 10.1007/s40003-011-0006-2

[8] Kumar A, Kroetsch T, Blondin P, Anzar M. Fertility-associated metabolites in bull seminal plasma and blood serum: 1H nuclear magnetic resonance analysis. Mol Reprod Dev 2015;82:123–31. 10.1002/mrd.22450 25640164 10.1002/mrd.22450

[9] Jodar M, Soler-Ventura A, Oliva R. Semen proteomics and male infertility. J Proteomics 2017;162:125–34. 10.1016/j.jprot.2016.08.018 27576136 10.1016/j.jprot.2016.08.018

[10] Westfalewicz B, Dietrich MA, Mostek A, Partyka A, Bielas W, Niżański W, et al Identification and functional analysis of bull *(Bos taurus)* cauda epididymal fluid proteome. J Dairy Sci 2017;100:6707–19. 10.3168/jds.2016-12526 28551182 10.3168/jds.2016-12526

[11] Westfalewicz B, Dietrich MA, Mostek A, Partyka A, Bielas W, Niżański W, et al Analysis of bull *(Bos taurus)* seminal vesicle fluid proteome in relation to seminal plasma proteome. J Dairy Sci 2017;100:2282–98. 10.3168/jds.2016-11866 28041731 10.3168/jds.2016-11866

[12] Kelly VC, Kuy S, Palmer DJ, Xu Z, Davis SR, Cooper GJ. Characterization of bovine seminal plasma by proteomics. Proteomics 2006;6:5826–33. 10.1002/pmic.200500830 17001600 10.1002/pmic.200500830

[13] Moura AA, Koc H, Chapman DA, Killian GJ. Identification of proteins in the accessory sex gland fluid associated with fertility indexes of dairy bulls: A proteomic approach. J Androl 2006;27:201–11. 10.2164/jandrol.05089 16278371 10.2164/jandrol.05089

[14] Somashekar L, Selvaraju S, Parthipan S, Patil SK, Binsila BK, Venkataswamy MM, et al Comparative sperm protein profiling in bulls differing in fertility and identification of phosphatidylethanolamine-binding protein 4, a potential fertility marker. Andrology 2017;5:1032–51. 10.1111/andr.12404 28859251 10.1111/andr.12404

[15] Nynca J, Dietrich MA, Ciereszko A. DIGE Analysis of Fish Tissues. Methods Mol Biol 2018;1664:203–219. 10.1007/978-1-4939-7268-5_16 29019135 10.1007/978-1-4939-7268-5_16

[16] Tripathi UK, Aslam MMK, Pandey S, Nayak S, Chhillar S, Srinivasan A, et al Differential proteomic profile of spermatogenic and Sertoli cells from peri-pubertal testes of three different bovine breeds. Front Cell Dev Biol 2014;2 10.3389/fcell.2014.00024 25364731 10.3389/fcell.2014.00024 PMC4206989

[17] Westfalewicz B, Dietrich MA, Ciereszko A. Impact of cryopreservation on bull *(Bos taurus)* semen proteome. J Anim Sci 2015;93:5240–53. 10.2527/jas.2015-9237 26641044 10.2527/jas.2015-9237

[18] Kaya A, Memili E. Sperm macromolecules associated with bull fertility. Anim Reprod Sci 2016;169:88–94. 10.1016/j.anireprosci.2016.02.015 26925808 10.1016/j.anireprosci.2016.02.015

[19] O’Flaherty C, Matsushita-Fournier D. Reactive oxygen species and protein modifications in spermatozoa†. Biol Reprod 2017 10.1093/biolre/iox104 29025014 10.1093/biolre/iox104

[20] Aitken RJ, Baker MA. Oxidative stress and male reproductive biology. Reprod Fertil Dev 2004;16:581–8. doi: 10.10371/RD03089 15367373 10.10371/RD03089

[21] Shiva M, Gautam AK, Verma Y, Shivgotra V, Doshi H, Kumar S. Association between sperm quality, oxidative stress, and seminal antioxidant activity. Clin Biochem 2011;44:319–24. 10.1016/j.clinbiochem.2010.11.009 21145315 10.1016/j.clinbiochem.2010.11.009

[22] Aydemir B, Onaran I, Ali {, Kiziler R, Alici B, Can Akyolcu M. The Influence of Oxidative Damage on Viscosity of Seminal Fluid in Infertile Men. J Androl 2008;29:4146 10.2164/jandrol.107.003046 17673435 10.2164/jandrol.107.003046

[23] Mostek A, Dietrich MA, Słowińska M, Ciereszko A. Cryopreservation of bull semen is associated with carbonylation of sperm proteins. Theriogenology 2017;92:95–102. 10.1016/j.theriogenology.2017.01.011 28237350 10.1016/j.theriogenology.2017.01.011

[24] Mostek A, Słowińska M, Judycka S, Karol H, Ciereszko A, Dietrich MA. Identification of oxidatively modified proteins due to cryopreservation of carp semen. J Anim Sci 2018;in press. 10.2527/jas2017.1930 10.1093/jas/sky063 PMC6140970 29534196

[25] Flesch FM, Gadella BM. Dynamics of the mammalian sperm plasma membrane in the process of fertilization. Biochim Biophys Acta—Rev Biomembr 2000;1469:197–235. 10.1016/S0304-4157(00)00018-6 10.1016/s0304-4157(00)00018-6 11063883

[26] Karunakaran M, Devanathan TG. Evaluation of bull semen for fertility-associated protein, *in vitro* characters and fertility. J Appl Anim Res 2016;2119:1–9. 10.1080/09712119.2015.1129343

[27] Talevi R, Gualtieri R. Molecules involved in sperm-oviduct adhesion and release. Theriogenology 2010;73:796–801. 10.1016/j.theriogenology.2009.07.005 19682733 10.1016/j.theriogenology.2009.07.005

[28] Rodríguez-Villamil P, Hoyos-Marulanda V, Martins JAM, Oliveira AN, Aguiar LH, Moreno FB, et al Purification of binder of sperm protein 1 (BSP1) and its effects on bovine in vitro embryo development after fertilization with ejaculated and epididymal sperm. Theriogenology 2016;85:540–54. 10.1016/j.theriogenology.2015.09.044 26553567 10.1016/j.theriogenology.2015.09.044

[29] Samanta L, Swain N, Ayaz A, Venugopal V, Agarwal A. Post-Translational Modifications in sperm Proteome: The Chemistry of Proteome diversifications in the Pathophysiology of male factor infertility. Biochim Biophys Acta 2016;1860:1450–65. 10.1016/j.bbagen.2016.04.001 27062907 10.1016/j.bbagen.2016.04.001

[30] Morielli T, O’Flaherty C. Oxidative stress impairs function and increases redox protein modifications in human spermatozoa. Reproduction 2015;149:113–23. 10.1530/REP-14-0240 25385721 10.1530/REP-14-0240 PMC5489333

[31] Madian AG, Regnier FE. Proteomic identification of carbonylated proteins and their oxidation sites. J Proteome Res 2010;9:3766–80. 10.1021/pr1002609 20521848 10.1021/pr1002609 PMC3214645

[32] Desai N, Sabanegh E, Kim T, Agarwal A. Free Radical Theory of Aging: Implications in Male Infertility. Urology 2010;75:14–9. 10.1016/j.urology.2009.05.025 19616285 10.1016/j.urology.2009.05.025

[33] Agarwal A, Durairajanayagam D, Halabi J, Peng J, Vazquez-Levin M. Proteomics, oxidative stress and male infertility. Reprod Biomed Online 2014;29:32–58. 10.1016/j.rbmo.2014.02.013 24813754 10.1016/j.rbmo.2014.02.013

[34] Urner F, Sakkas D. Protein phosphorylation in mammalian spermatozoa. Reproduction 2003 10.1530/rep.0.1250017 10.1530/rep.0.1250017 12622692

[35] Chen Y, Liang P, Huang Y, Li M, Zhang X, Ding C, et al Glycerol kinase-like proteins cooperate with Pld6 in regulating sperm mitochondrial sheath formation and male fertility. Cell Discov 2017;3 10.1038/celldisc.2017.30 28852571 10.1038/celldisc.2017.30 PMC5566117

[36] Chakrabarti R, Cheng L, Puri P, Soler D, Vijayaraghavan S. Protein phosphatase PP1 gamma 2 in sperm morphogenesis and epididymal initiation of sperm motility. Asian J Androl 2007;9:445–52. 10.1111/j.1745-7262.2007.00307.x 17589781 10.1111/j.1745-7262.2007.00307.x

[37] Pereira R, Sá R, Barros A, Sousa M. Major regulatory mechanisms involved in sperm motility. Asian J Androl 2015;19:5–14. 10.4103/1008-682X.167716 26680031 10.4103/1008-682X.167716 PMC5227674

[38] O’Flaherty C. Redox regulation of mammalian sperm capacitation. Asian J Androl 2015;17:583–90. 10.4103/1008-682X.153303 25926608 10.4103/1008-682X.153303 PMC4492048

[39] Flores E, Fernández-Novell JM, Peña A, Rodríguez-Gil JE. The degree of resistance to freezing-thawing is related to specific changes in the structures of motile sperm subpopulations and mitochondrial activity in boar spermatozoa. Theriogenology 2009;72:784–97. 10.1016/j.theriogenology.2009.05.013 19604570 10.1016/j.theriogenology.2009.05.013

[40] Nolan M, Babcock DF, Wennemuth G, Brown W, Burton K a, McKnight GS. Sperm-specific protein kinase A catalytic subunit Calpha2 orchestrates cAMP signaling for male fertility. Proc Natl Acad Sci U S A 2004;101:13483–8. 10.1073/pnas.0405580101 15340140 10.1073/pnas.0405580101 PMC518783

[41] Agarwal A, Virk G, Ong C, du Plessis SS. Effect of Oxidative Stress on Male Reproduction. World J Mens Health 2014;32:1–17. 10.5534/wjmh.2014.32.1.1 24872947 10.5534/wjmh.2014.32.1.1 PMC4026229

[42] Dun MD, Aitken RJ, Nixon B. The role of molecular chaperones in spermatogenesis and the post-testicular maturation of mammalian spermatozoa. Hum Reprod Update 2012;18:420–35. 10.1093/humupd/dms009 22523110 10.1093/humupd/dms009

[43] Hemachand T, Gopalakrishnan B, Salunke DM, Totey SM, Shaha C. Sperm plasma-membrane-associated glutathione S-transferases as gamete recognition molecules. J Cell Sci 2002;15:2053–65. 10.1242/jcs.115.10.2053 11973347

