# Supplementary figures and images for: Differences in sperm protein abundance and carbonylation level in bull ejaculates of low and high quality
Source: PLoS One. 2018 Nov 14;13(11):e0206150. doi: 10.1371/journal.pone.0206150 (PMC6241115; doi:10.1371/journal.pone.0206150)

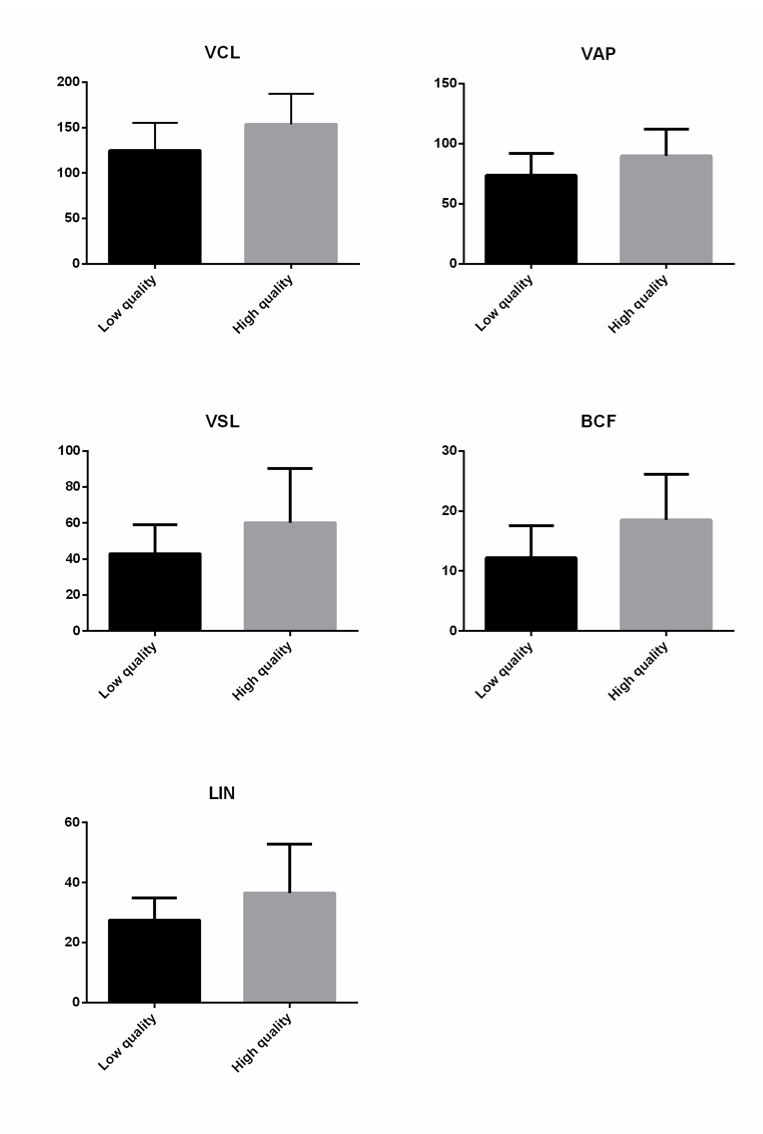

Supplement: S1 Fig — Results are expressed as the mean +SD. No significant differences in the movement trajectory were found between LQ and HQ semen. VCL − curvilinear velocity; VAP − average path velocity; VSL − straight line velocity; BCF − beat cross frequency; and LIN − linearity. (TIF) [file pone.0206150.s001.tif]

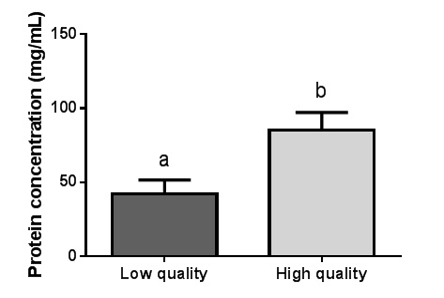

Supplement: S2 Fig — Results are expressed as the mean +SD. Different letters indicate significant differences (p < 0.05). (TIF) [file pone.0206150.s002.tif]
